# Nanocomposite Co_3_O_4_-ZnO Thin Films for Photoconductivity Sensors

**DOI:** 10.3390/s23125617

**Published:** 2023-06-15

**Authors:** Victor V. Petrov, Victor V. Sysoev, Irina O. Ignatieva, Irina A. Gulyaeva, Maria G. Volkova, Alexandra P. Ivanishcheva, Soslan A. Khubezhov, Yuri N. Varzarev, Ekaterina M. Bayan

**Affiliations:** 1 Institute of Nanotechnologies, Electronics, and Equipment Engineering, Southern Federal University, Taganrog 347922, Russia; vvpetrov@sfedu.ru (V.V.P.); iten@sfedu.ru (I.A.G.); starnikova@sfedu.ru (A.P.I.); soslan.khubezhov@gmail.com (S.A.K.); varzarevyuv@sfedu.ru (Y.N.V.); 2 Institute of Physics and Technology, Yuri Gagarin State Technical University of Saratov, Saratov 410054, Russia; 3 Faculty of Chemistry, Southern Federal University, Rostov-on-Don 344090, Russia; iignateva@sfedu.ru (I.O.I.); ekbayan@sfedu.ru (E.M.B.); 4 Core Shared Research Facility «Physics and Technology of Nanostructures», North-Ossetian State University, Vladikavkaz 362025, Russia; 5 Department of Physics and Engineering, ITMO University, St. Petersburg 197101, Russia

**Keywords:** ZnO, Co_3_O_4_, metal oxide, nanocomposites, thin film, pyrolysis, heterojunction, photovoltaic sensors

## Abstract

Thin nanocomposite films based on zinc oxide (ZnO) added with cobalt oxide (Co_3_O_4_) were synthesized by solid-phase pyrolysis. According to XRD, the films consist of a ZnO wurtzite phase and a cubic structure of Co_3_O_4_ spinel. The crystallite sizes in the films increased from 18 nm to 24 nm with growing annealing temperature and Co_3_O_4_ concentration. Optical and X-ray photoelectron spectroscopy data revealed that enhancing the Co_3_O_4_ concentration leads to a change in the optical absorption spectrum and the appearance of allowed transitions in the material. Electrophysical measurements showed that Co_3_O_4_-ZnO films have a resistivity up to 3 × 10^4^ Ohm∙cm and a semiconductor conductivity close to intrinsic. With advancing the Co_3_O_4_ concentration, the mobility of the charge carriers was found to increase by almost four times. The photosensors based on the 10Co-90Zn film exhibited a maximum normalized photoresponse when exposed to radiation with wavelengths of 400 nm and 660 nm. It was found that the same film has a minimum response time of ca. 26.2 ms upon exposure to radiation of 660 nm wavelength. The photosensors based on the 3Co-97Zn film have a minimum response time of ca. 58.3 ms versus the radiation of 400 nm wavelength. Thus, the Co_3_O_4_ content was found to be an effective impurity to tune the photosensitivity of radiation sensors based on Co_3_O_4_-ZnO films in the wavelength range of 400–660 nm.

## 1. Introduction

Semiconductor oxide materials are commonly employed in almost all areas of modern industry. One of these environmentally friendly materials is zinc oxide, an n-type semiconductor with a wide band gap of 3.37 eV [1]. The combination of relative low cost and unique physicochemical properties makes zinc-oxide-based materials very attractive for research and various applications [2]. For example, ZnO films with the addition of aluminum oxide are optically transparent in the visible light range, which allows one to use these films in various optical devices [3,4]. The creation of composites based on ZnO and a small amount of other oxide leads to a change in the band gap of the material, which makes it possible to adjust the electrophysical properties of synthesized materials for use in optical, energy storage, and photovoltaic devices. Particularly, widespread additives for ZnO are p-type semiconducting oxides, such as NiO, CuO, Co_3_O_4_, etc., which lead to the appearance of local p-n junctions in the whole structure. Spinel Co_3_O_4_ is considered to be the most promising one of the listed oxides. Normally, this material exists as a mixed oxide of Co^3+^ and Co^2+^, characterized by the presence of an indirect and a double band gap of 2.2 eV and 1.6 eV [5]. It is assumed that this feature provides a photoresponse in a wide range of wavelengths corresponding to a fundamental absorption. The choice of Co_3_O_4_ as an efficient additive to ZnO has been confirmed by various studies, including, for instance, the study of zinc oxide films modified with iron, manganese, cobalt, or nickel ions [6]. It was noted that the largest narrowing of the band gap down to 3.13 eV was observed in ZnO containing 5 wt.% of cobalt. It was demonstrated that an oxide p-n junction of the Co_3_O_4_/ZnO can operate as a photodetector in the visible light range [7]. 

For successful application, Co_3_O_4_-ZnO films must be nanocrystalline and accounting for mass-scale production, and also the synthesis method must be simple, economical, and environmentally friendly to allow for cyclic production. In the literature, numerous protocols have been reported for deriving Co_3_O_4_-ZnO films, such as plasma-chemical vapor deposition (PE-CVD), chemical precipitation from solutions [8,9], laser deposition [5], sol-gel [10,11], solvothermal [12], electrochemical [13], hydrothermal [14], etc. However, most of these methods require expensive equipment, are time-consuming and energy-intensive procedures. Frequently, the quality of the required films is difficult to ensure regarding the many noted technologies. Still, the sol-gel method is often considered as the most efficient one, which makes it possible to properly introduce cobalt into the wurtzite structure [10]. Such Co inclusion to ZnO results in narrowing the band gap from 3.22 eV for pristine zinc oxide down to 2.39 eV upon adding 5 mol.% cobalt. The spray-coating technique also yields a hexagonal wurtzite structure of ZnO doped with Co^2+^ [15]. At the same time, it was shown in the literature [16] that increasing the concentration of Co from 1 at.% up to 4 at.% in the ZnO structure forces its band gap to extend from 3.3 eV to 3.52 eV. It is also known that the band gap study of Co_3_O_4_-ZnO films by optical methods should be carried out extremely carefully, because the presence of Co^2+^ and Co^3+^ ions in the spinel structure leads to the existence of several possible energy transitions and, in fact, to a decrease in the band gap [17,18]. Another interesting fact is the formation of p-n junctions in the structure of the Co_3_O_4_-ZnO film with the corresponding appearance of internal electric fields [19].

As shown in our previous studies, transparent, homogeneous, nanocrystalline films based on zinc oxide [20], as well as ZnO-SnO_2_ composite films [21,22], could be reliably obtained by solid-phase pyrolysis. In the framework of this method, we fabricated here the composite Co_3_O_4_-ZnO films whose properties are sufficiently tuned by the composition to be different from those of pure Co_3_O_4_ and ZnO ones. For example, studies of photoconductivity under the influence of constant radiation from a light-emitting diode (LED) at a 400 nm wavelength have ensured that the photoresponse time constant is quite large, more than 25 s, which is not typical for Co_3_O_4_ films [23]. In this manuscript, we study in detail the surface, optical, and electrophysical properties of nanocomposite Co_3_O_4_-ZnO films with a cobalt content in the range of 1–10 mol.% prepared by solid-phase pyrolysis, and also investigate their photoelectric properties under pulsed radiation exposures.

## 2. Materials and Methods

### 2.1. Materials

Cobalt acetate tetrahydrate (Co(CH_3_COO)_2_·4H_2_O), zinc acetate dihydrate (Zn(CH_3_COO)_2_·2H_2_O), abietic acid, and 1,4-dioxane as a solvent were employed as precursors for the synthesis of Co_3_O_4_-ZnO thin films. All chemicals used were of analytical grade or of the highest purity available.

### 2.2. Synthesis of Co_3_O_4_-ZnO Thin Films

The synthesis of transparent Co_3_O_4_-ZnO thin films was carried out in two stages according to an earlier reported protocol [22]. At the first stage, zinc(II) and cobalt(II) abietates were obtained from a melt of abietic acid and zinc(II) and cobalt(II) acetates. The molar ratios of cobalt and zinc were Co:Zn = 1:99 (sample 1Co-99Zn), 3:97 (sample 3Co-97Zn), 5:95 (sample 5Co-95Zn), and 10:90 (sample 10Co-90Zn) mol.%, respectively. The resulting melt was cooled and crushed. At the second stage, a mixture of zinc (II) and cobalt (II) abietates was dissolved in an organic solvent and applied to previously prepared substrates. To provide a comprehensive study of the properties, substrates with various properties, such as sodium-calcium-silicate glass, quartz glass, Al_2_O_3_, and Si/SiO_2_, were utilized. The application of the solution to the substrate was carried out three times in row to ensure the required film thickness. Each layer was dried at 100 °C. The final heat treatment was carried out in a muffle furnace at 600 °C for 2 h. For comparison, films of pristine zinc oxide were also synthesized by the same technology route.

### 2.3. Materials’ Characterization

The materials under study were characterized via X-ray diffraction (XRD) with the help of an ARLX’TRA diffractometer (Thermo Scientific, Ecublens, Switzerland) of CuK_α_ source with radiation at a wavelength of λ = 0.15406 nm in the 20–80° range. Electron microscopy (SEM) was employed to reveal the surface morphology with a Nova Nanolab 600 microscope (FEI Company, Eindhoven, The Netherlands); the electrons were accelerated with 10 keV energy. The material transformation under heating was revealed under thermogravimetric analysis (TGA) and differential scanning calorimetric (DSC) techniques in a Jupiter Jupted thermal analyzer (Netzsch—Geratebau GmbH, Selb, Germany) under air atmosphere in a 298–1173 K temperature range with a heating rate of 10 K/min. The optical absorption and transmission spectra were recorded with a UV-1100 ECOVIEW spectrophotometer (Shanghai, China) at a 300–1000 nm wavelength range.

X-ray photoelectron spectroscopy (XPS) was applied to study the surface composition and chemical state of materials with a K–Alpha Thermo Scientific spectrometer (Thermo Fisher Scientific, Oxford, UK) and an Al_Kα_ radiation source at 1486.6 eV energy, according to earlier reported protocols [24,25,26]. Upon measurements, the samples were placed into a vacuum chamber at pressure below 2∙10^−9^ mbar. To suppress the surface charging, a flood gun was utilized. The XPS survey spectra were collected at the constant energy of −200 eV under 1 eV resolution. The intensities were integrated to account for 10 sequential recordings. The high-resolution XPS spectra were measured under the transmission energy of 20 eV at 0.1 eV spectrum resolution; the number of scans was equal to 20. The sample spot was 400 μm in diameter. To analyze the curves, they were fitted with Shirley and Tugard functions; a linear shape of the constructed curves was obtained at a 30% Lorentz/Gauss mixture ratio [27,28,29].

For ensuring electrophysical measurements and form sensors, V–Ni contact strips, 200 nm thick, were screen-printed by thermal vacuum technique over the films under study. The electrical conductance, G, dependence on temperature, T, and current-voltage characteristics (I-V) were studied with the help of a setup described earlier elsewhere [30]. Taking G(T) curves, we could evaluate the activation energy, E_a_, via the Arrhenius equation as follows [31]:

G = G_0_ exp(−E_a_/k∙T) (1)

where k is the Boltzmann constant and G_0_ is the coefficient taking the bulk material conductivity into account.

The semiconductor parameters, concentration, and mobility of charge carriers were estimated by Hall effect measurements, taking the corresponding setup (ECOPIA HMS-3000, Ecopia Corporation, Anyang city, Republic of Korea) at 0.59 T of a magnetic field at temperatures ranging from room up to 300 °C. For this purpose, the materials under study were placed over square Al_2_O_3_ substrates that measured 10 × 10 mm^2^. 

The surface morphology and potential in the materials under study were evaluated in the Ntegra probe nanolab (NT-MDT SI, Moscow, Russia) with atomic force microscopy (AFM) and Kelvin probe force microscopy (KPFM) techniques. To record the data, a cantilever of 25 nm curvature was applied under the force constant of 11.2 N/m. The KPFM data were processed by Image Analysis software (NT-MDT, Moscow, Russia) to obtain the maximum values of the surface potential, V_B_. The V_B_ values were averaged over each sample following the performance of a few scans.

### 2.4. Photovoltaic Measurements

The earlier developed setup [23] allows us to measure the photoconductivity kinetics of resistive structures based on the film under study upon irradiation with an LED at a given wavelength. We selected several LEDs (GNL type, Zhuangshi, China) having maximum emissions at 660 nm and 400 nm wavelengths. The radiation LED intensity was adjusted by a power supply (AKIP-1101, Manson Engineering Industrial Ltd., Kwai Chung, China), while the photoconductivity was controlled by a digital multimeter (Tektronix DMM 4050, Beaverton, OR, USA). 

Studies of the samples under a pulsed irradiation were carried out by measuring the time dependences of the photocurrent following the pulses of the light; the same LEDs, of 400 nm and 660 nm wavelengths, were taken for these measurements. The pulses of light illumination were applied to the samples with On/Off pulses of voltage feeding the LEDs driven by an Aktakom APS-7303 power supply (Aktakom, Moscow, Russia); the illumination pulse time was 0.1 s. The current measurements were carried out with a Keithley 6487 Picoammeter/Voltage Source (Keithley Instruments, Inc., Cleveland, OH, USA) under applying the bias of 40 V to be fed to the oscilloscope (LeCroy WP 7100A, LeCroy Corporation, Chestnut Ridge, NY, USA). The oscilloscope time scan was synchronized with the pulsing of LED irradiation. All the units were controlled by a personal computer (PC) driven by home-made software composed in MATLAB software (The MathWorks, Inc., Natick, MA, USA).

## 3. Results and Discussion

### 3.1. TGA and DSC

**Figure 1:**
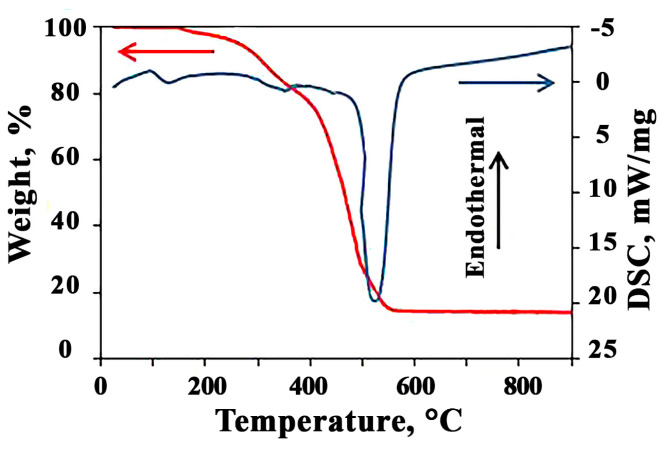
TGA and DSC for intermediates of 10Co-90Zn.

To find out the optimal temperature of the synthesis process, thermogravimetric analysis and differential scanning calorimetry of intermediates obtained at the first stage of synthesis were carried out (Figure 1).

As one can see from the figure, when heating a mixture of abietates in the temperature range from room temperature up to 900 °C, several regions can be distinguished on the TG-DTA curve. At the first temperature interval of 25–115 °C, the adsorbed water is removed. This process has a positive value of the thermal effect. Following the further temperature evolving up to 420 °C, we observed a gradual thermal decomposition of organic zinc and cobalt salts, also accompanied by heat absorption. When reaching the temperature equal to 530 °C, there is a sharp modification in the mass of the intermediate material with a release of a large amount of energy, as clearly seen with the peak on the DTA curve. This exothermic effect is due to the formation of new bonds in the wurtzite phase under crystallization of the ZnO oxide film. When the temperature is higher than 570 °C, the mass of the material has been stabilized. The total weight loss is 86%. These data are in a good agreement with the proposed process of the oxidation of metal abietates with the appearance of the oxide phase. From the obtained data, the temperature for the oxide calcination equal to 600 °C was justified.

### 3.2. XRD

**Figure 2:**
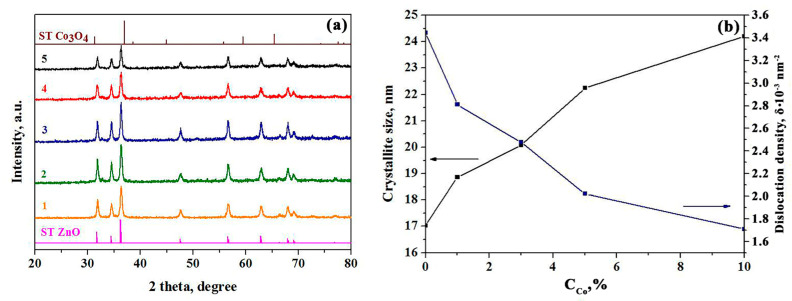
XRD patterns of pure ZnO (1), 1Co-99Zn (2), 3Co-97Zn (3), 5Co-95Zn (4), and 10Co-90Zn (5) (**a**) and dependences of the crystallite size and dislocation density on the Co_3_O_4_ (**b**) content.

Figure 2 shows typical XRD patterns of synthesized Co_3_O_4_-ZnO materials containing various cobalt loadings to be calcined at 600 °C. The major phase is the hexagonal structure of ZnO wurtzite (ICSD No. 65119 [32]) regardless of the additive concentration. For the 5Co-95 Zn and 10Co-90 Zn composites we observed peaks corresponding to (113), (044), and (115) crystal planes, which confidently characterize the growing of a cubic spinel Co_3_O_4_ structure (ICSD Card No. 063165 [33]). Thus, the XRD data confirm forming a composite material of the desired content. No other phases were found in the synthesized materials, which indicates the purity of the obtained material. The XRD data are in good agreement with the elemental composition obtained as a result of the XPS analysis.

The particle size was estimated with the Scherrer equation. According to calculations, all materials are characterized by the presence of a nanogranular structure (Figure 2b), where the crystal sizes range from 17–24 nm. It is clear that increasing the cobalt oxide content in the film forces enlarging the particle size. The dislocation density, δ, was calculated by Formula (2) [34]:

δ = 1/D^2^ (2)

where D is the particle size.

We plotted these data in Figure 2 as well. As one can see, the higher content of cobalt oxide in Co_3_O_4_–ZnO films yields lower dislocation density. Obviously, this effect is due to increasing the particle sizes to be accompanied with reducing defects in the film. This trend is generally consistent with data observed in other research [35,36,37].

### 3.3. SEM

**Figure 3:**
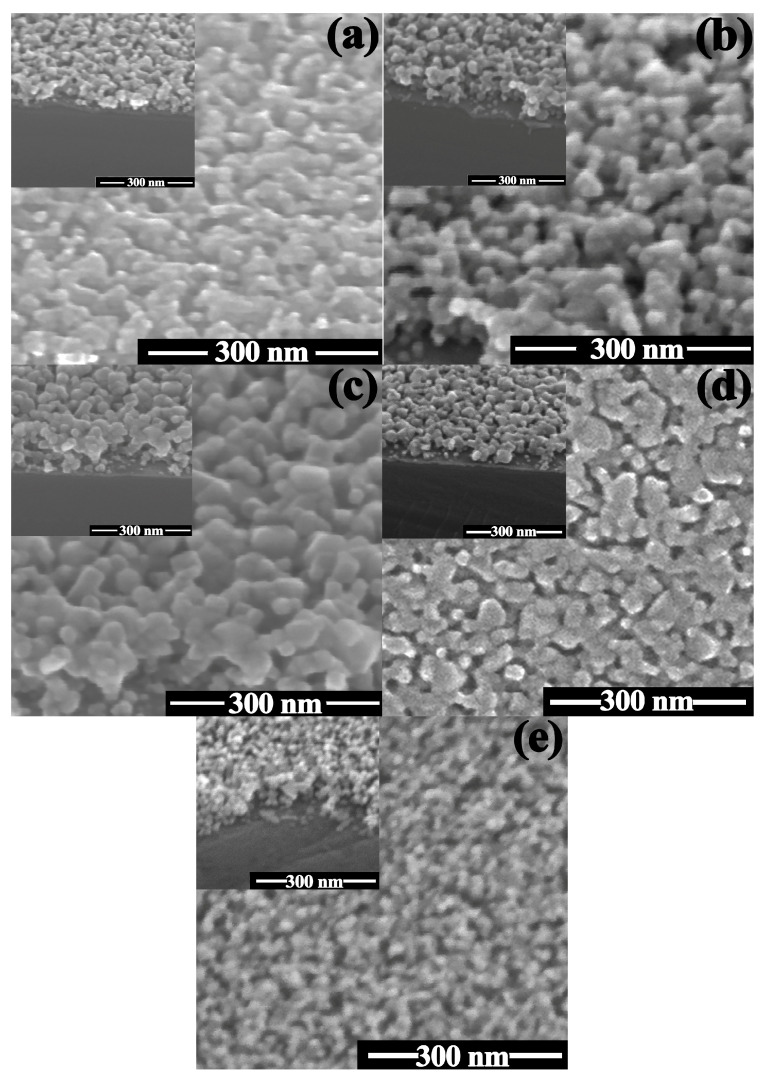
SEM photos of composites under study: (**a**) 1Co-99 Zn; (**b**) 3Co-97 Zn; (**c**) 5Co-95 Zn; (**d**) 10Co-90 Zn; (**e**) pure ZnO.

The 1Co-99Zn, 3Co-97Zn, 5Co-95Zn, 10Co-90Zn, and pure ZnO films, calcined at 600 °C, were inspected with SEM; the typical images are given in Figure 3. It can be observed that smaller particles of ZnO form a coating where we could distinguish large particles of Co_3_O_4_. For 5Co-95 Zn and 10Co-90 Zn composites, the appearance of a large number of agglomerated particles is observed. According to SEM data, the films thickness is 150–200 nm [23]. The calculation of the particle sizes composing the films was carried out using a statistical analysis of SEM images to compare with the XRD estimations of particle size. It was found that the particle size increases with the increase in cobalt oxide concentration. These data correlate well with similar results obtained from XRD patterns via the Scherrer equation. The particle sizes forming the film were statistically calculated for 150 particles according to the software Digimizer 4.1.1.0. The error in determining the crystallite sizes in this program can be defined as ±2 nm. For all Co_3_O_4_-ZnO films, the crystallite sizes were in the range of 10–36 nm. However, the average size of the crystallites for each film was different. The variance estimate was ±4 nm. Therefore, for the material 1Co-99Zn, the average crystallite size is 17 ± 4 nm, for the material 3Co-97Zn is 23 ± 4 nm, for the material 5Co-95Zn is 25 ± 4 nm, and for the material 10Co-90Zn is 20 ± 4 nm (Figure 3).

### 3.4. Optical Properties

**Figure 4:**
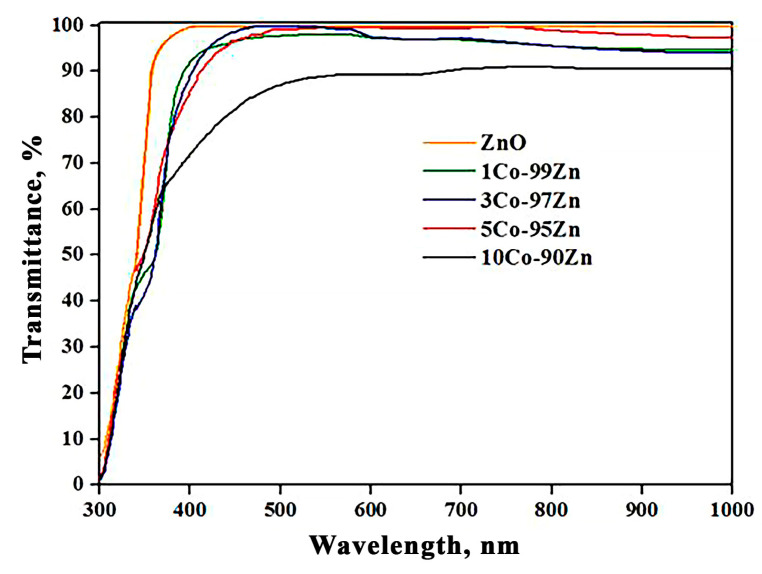
Optical transmittance spectra of Co_3_O_4_-ZnO films of various composition.

The analysis of transmission spectra recorded with the films under study is given in Figure 4. It has been noted that Co_3_O_4_-ZnO films of 1Co-99Zn, 3Co-97Zn, and 5Co-95Zn composition transmit more than 80% of the radiation in the visible light range. With the introduction of cobalt oxide, the transmission goes down and reaches more than 65% for the 10Co-90Zn sample in the visible light range. The maximum transmission is 90% in the range of 550–1000 nm wavelengths.

**Figure 5:**
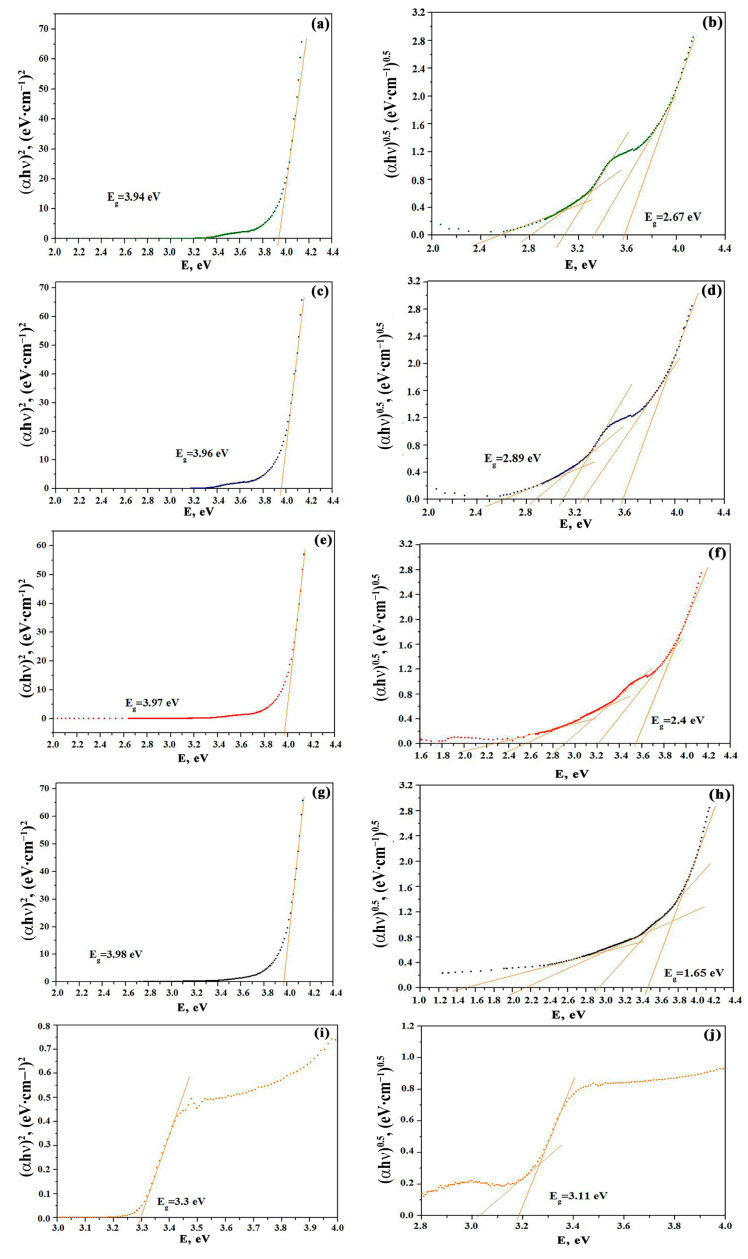
Evaluation of the band gap of the composites under study: (**a**,**b**) 1Co-99Zn; (**c**,**d**) 3Co-97Zn; (**e**,**f**) 5Co-95Zn; (**g**,**h**) 10Co-90Zn; (**i**,**j**) pure zinc oxide.

The band gap, E_g_, was estimated for all the materials under study taking the Tauc plots as the dependence of (αhν)^2^ on the photon energy. The data are collected in Figure 5. In another work [38], it was shown that films of pure ZnO obtained by solid-phase pyrolysis have a band gap of 3.30 eV. This value is somewhat lower than the band gap of single-crystal zinc oxide due to the influence of the presence of nanocrystals [22,39]. By extrapolating the direct part of the (αhν)^2^ curve to the point of α = 0, it was found that the band gaps for the films 1Co-99Zn, 3Co-97Zn, 5Co-95Zn, and 10Co-90Zn are 3.94, 3.96, 3.97, and 3.98, respectively (Figure 5). In the Tauc plots, it can also be noticed that the extrapolation line changes its slope to be increased with a higher cobalt oxide concentration (Figure 5a,c,d,e). In addition, the curves show the presence of a sufficiently large Urbach tail. This is due, on the one hand, to the nanoscale film structure, which leads to greater absorption for energies below the band gap [22,39]. On the other hand, this may be due to the presence of surface electric fields [21,22]. In the structure of Co_3_O_4_-ZnO films, surface electric fields can exist because crystallites of p- and n-type semiconductors are in contact [19]. In addition, Co_3_O_4_ films are characterized by the presence of two absorption edges. This can be clearly seen in the Tauc plots of Figure 5b,d,f,g, which allow us to see additional energy levels that contribute to indirect transitions with lower energy. Using the technique described earlier in [22,31], it can be seen that the energy of indirect transitions for the ZnO film is 3.11 eV, which does not differ so much from the band gap of 3.3 eV. At the same time, the extrapolation of individual sections of the Tauc plot indicates that there are indirect transitions, with energies significantly lower than the band gap found from the curves of (αhν)^2^ on the photon energy. Therefore, for the 1Co-99Zn composite, for example, these values are 2.67, 2.94, and 3.45 eV. The same values for the 3Co-97Zn sample are 2.63, 2.90, and 3.42 eV, for 5Co-95Zn are 2.42, 2.72, and 3.38, and for 10Co-90Zn are 1.65, 2.54, and 3.20 eV, respectively.

### 3.5. Electrophysical Properties

**Figure 6:**
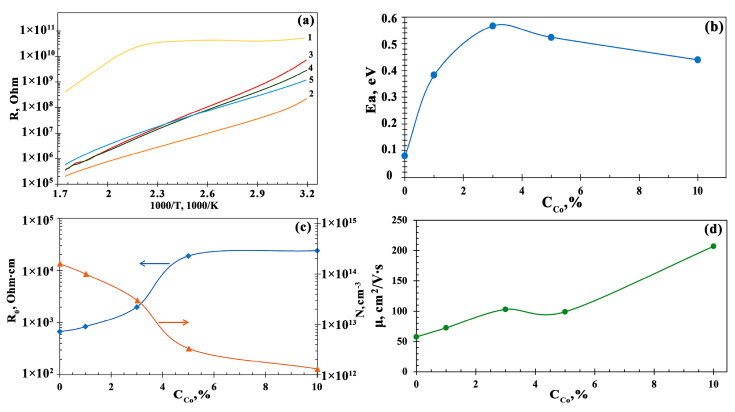
Electrophysical characteristics of Co_3_O_4_–ZnO composites under study: (**a**) dependence of resistance on the reverse temperature of ZnO (1), 1Co-99Zn (2), 3Co-97Zn (3), 5Co-95Zn (4), and 10Co-90Zn (5) films; (**b**–**d**) dependence of activation energy of conductivity (**b**), concentration of charge carriers (N) and resistivity (ρ) (**c**), and the mobility of charge carriers (μ) (**d**) on the concentration of cobalt in the composite films.

To estimate the activation energy of conductivity in thin Co_3_O_4_-ZnO films, the temperature dependences of the film conductivity in the range from room temperature to 300 °C were measured; the data are present in Figure 6a. Further, using the Arrhenius equation, the activation energy of conductivity (E_a_) values were calculated as given in Figure 6b. In addition, the concentrations of charge carriers, N, resistivity, ρ, and mobility of charge carriers, μ, were measured by the Hall effect method (Figure 6).

An analysis of measurement results presented in Figure 6 shows that the addition of Co_3_O_4_ crystallites, which are p-type semiconductors, to a nanoscale n-type semiconductor, ZnO, leads to reducing both the concentration of charge carriers and resistivity by two orders of magnitude. This is explained by a recombination of electrons, which are the major charge carriers in ZnO and holes, which are the major charge carriers in Co_3_O_4_. At the same time, with the increase in cobalt oxide concentration in the Co_3_O_4_-ZnO films to 3%, the activation energy of conductivity increases more than seven times (from 0.08 eV to 0.57 eV). If the concentration of cobalt oxide increases from 3% to 10%, E_a_ decreases only by 25% (from 0.57 eV to 0.44 eV). This behavior of the activation energy of conductivity in Co_3_O_4_-ZnO films with an increase in the cobalt oxide concentration is associated with the decrease in charge carrier concentration due to their recombination, as evidenced by the graph in Figure 6c: electrons with lower activation energy recombine at concentrations of cobalt oxide in films up to 3%. With an increase in the concentration of cobalt oxide above 3–5%, the values of E_a_ and N change slightly. Due to the low concentration of charge carriers, the Co_3_O_4_-ZnO composite can be considered to be an intrinsic semiconductor. At the same time, nanocrystallites of p- and n-type semiconductors are in close contact in this material. This leads to the existence of strong internal electric fields. The presence of such fields contributes to advancing the mobility of charge carriers by two or more times with the higher content of Co_3_O_4_ in the composite films.

### 3.6. XPS Analysis

The analysis of elemental and chemical composition was carried out in an ultra-high vacuum of pressure below 1.8 × 10^−9^ mbar. Before conducting the research, the following reference values of the photoelectronic lines for reference samples were derived as Au 4f—84 eV, Ag 3d—368.2 eV, and Cu 2p—932.6 eV. A compensation gun was used to neutralize the surface charge. The value of the binding energy for the carbon photoelectronic line of C 1s was BE = 284.8 eV.

**Figure 7:**
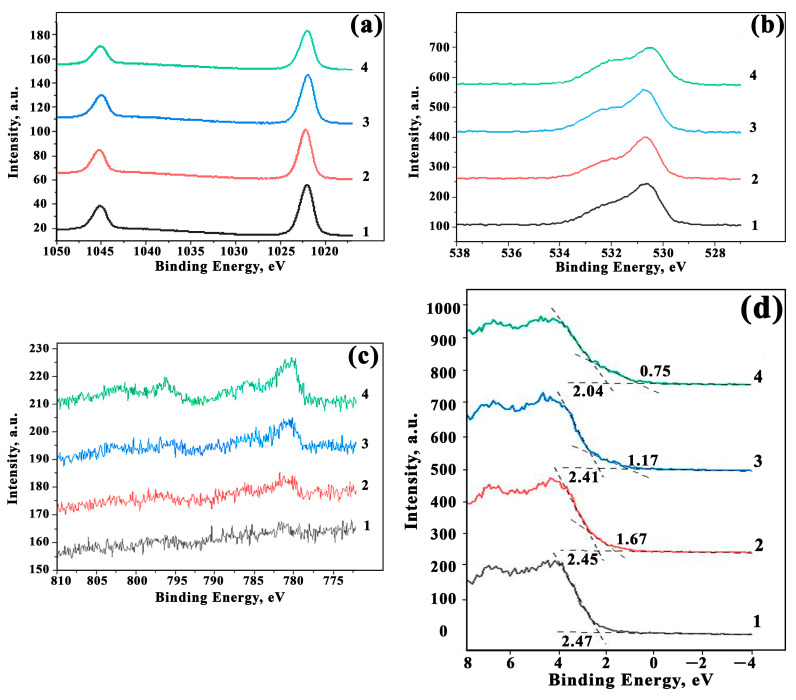
XPS characteristics of Co_3_O_4_-ZnO composites under study: (**a**) high-resolution XPS spectra of Zn2p; (**b**) high-resolution XPS spectra of O1s; (**c**) high-resolution XPS spectra of Co2p; (**d**) the maximum valence band maximum for 1Co-99Zn (1), 3Co-97Zn (2), 5Co-95Zn (3), and 10Co-90Zn (4) films.

The elemental composition of the composite films was determined from the high-resolution spectra of C1s, O1s, Zn2p, and Co2p photoelectronic lines obtained in the mode of constant transmission energy with a pass energy equal to 20 eV and a spectral resolution of 0.1 eV from the surface area of 400 μm; the number of scans was N = 10. The component analysis was carried out by constructing curves using joint Shirley and Tugard functions to define the peak background, while the shape of the curves follows the integration of Lawrence functions/Gaussian = 30%. Figure 7 shows high-resolution spectra for the Co_3_O_4_-ZnO films under study.

Figure 7a shows that the shape and positions of Zn2p spectra photoelectronic lines around 1021.4 eV indicate that zinc oxide exists in an oxidized state. This is also evidenced by the photoelectronic peak of O1s at 530.4 eV present in Figure 7b. The spectrum of oxygen, O1s, has an asymmetric shape to be described by two components. The major peak at 530.4 eV corresponds to the lattice oxygen anions in the pure ZnO. The second peak at 531.7 eV matures from the presence of various particles with C-O and C=O bonds. These carbon species seem to be present due to the film synthesis method from organic precursors and from the surface upon adsorption. The increase in cobalt content in the nanocomposites does not lead to a change in the O1s spectrum. The spectra of Co2p are drawn in Figure 7c. It can be seen that the experimental curves can be described by two doublets corresponding to Co^3+^ at 780.0 eV and Co^2+^ at 782.3 eV. The latter one corresponds to the appearance of the cobalt oxide as Co_3_O_4_.

Taking the data of the photoelectron spectra, we calculated the ratio of elements in Co_3_O_4_-ZnO films, as listed in Table 1. These data confirmed that the content of cobalt in the film corresponds well to that in the precursor.

Modifying the surface of nanocomposite oxides can lead to changes in the electron work function. Taking a valence band maximum (VBM) can be used to clarify the position of the Fermi level at the surface and evaluate the band gap in semiconductors [40,41]. Figure 7d shows the photoelectronic lines of the maximum valence band (E_VB_) and their values, and also that the higher Co concentrations result in lower E_VB_ values. A particularly strong reduction is observed for the 10Co-90Zn film. Knowing the values of the activation energy, which are shown in Figure 7b, the band gaps are estimated to be equal to 2.85 eV, 3.02 eV, 2.93 eV, and 2.48 eV for films of 1Co-99Zn, 3Co-97Zn, 5Co-95Zn, and 10Co-90Zn, respectively. In addition, the approximation of the XPS spectra indicates the presence of additional energy levels matured from the presence of Co_3_O_4_ crystallites in the films. For 1Co-99Zn, 3Co-97Zn, 5Co-95Zn, and 10Co-90Zn films, the additional levels have energies of 2.05 eV, 2.24 eV, 1.59 eV, and 1.19 eV, respectively. This is partly consistent with the results obtained from the Tauc curves. The straightening of the Tauc curves in the coordinates α^1/2^ vs. hν shows that indirect transitions of charge carriers are present in [31]. At the same time, some energy states are not reflected in the Tauc curves. This is due to the fact that the concentration of charge carriers in the resulting material is low (≤10^14^ cm^−3^). In this case, the impurity atoms do not interact with each other and form narrow levels in the band gap with the formation of charge carrier transitions with different energies [31]. In addition, the transition energy in the range of 1.8–2.2 eV can correspond to the transitions of charge carriers between O^2−^ and Co^2+^, and the transition energy of 1.5–1.6 eV corresponds to the charge transfer between O^2−^ and Co^3+^ [18]. Transitions of about 1 eV correspond to the charge transfer between Co^2+^ and Co^3+^ [5].

### 3.7. Surface Properties

**Figure 8:**
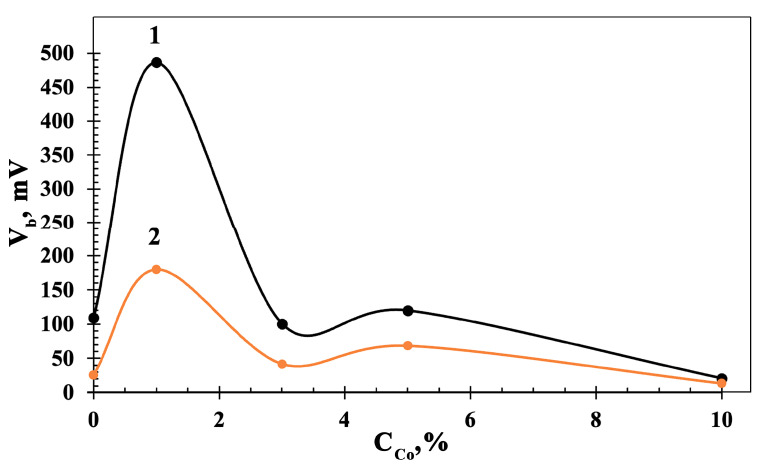
Dependence of the maximum (1) and average (2) values of surface potential (V_b_) on the concentration of Co_3_O_4_ in Co_3_O_4_-ZnO films.

The morphology and distribution of the surface potential V_b_ in Co_3_O_4_-ZnO films was studied by AFM and KPFM. Upon the measurements, the first pass of the surface cantilever was considered in semi-contact mode to primarily measure the relief profile, while the second pass allowed us to evaluate the surface potential by guiding the cantilever at a fixed distance from the surface. The data are collected in Figure S1 (Supporting File) and Figure 8. The KPFM measurements showed that the highest maximum and average V_b_ values, of 487 mV and 180 mV, respectively, are characteristic for the 1Co-99Zn film. For the 3Co-97Zn and 5Co-95Zn films, the maximum values of V_b_ were 60 mV and 120 mV, respectively, while the average values were 1.5–2 times less. Such high values of the surface potential indicate the presence of a surface electric field with the strength reaching values of 10^5^–10^7^ V/cm [21,22]. The minimum average value of the surface potential of 13.8 mV was observed for the 10Co-90Zn material. In the 1Co-99Zn films, where the crystallite sizes are the smallest (Figure 2b), ZnO crystallites surround Co_3_O_4_ crystallites, yielding local p-n transitions. Therefore, there is a strong depletion of Co_3_O_4_ crystallites by the major free charge carriers. This leads to large values of V_b_. With increasing the concentration of Co_3_O_4_ crystallites, the crystallite sizes enlarge, which lowers the depletion of Co_3_O_4_ crystallites; therefore, the surface potential reduces. The higher content of Co_3_O_4_ in the films also leads to decreasing the concentration of free charge carriers and increases both the resistivity and mobility of the carriers. We also observed a similar phenomenon in ZnO-SnO_2_ nanocomposite films prepared by solid-phase pyrolysis [21].

### 3.8. Photoconductivity

**Figure 9:**
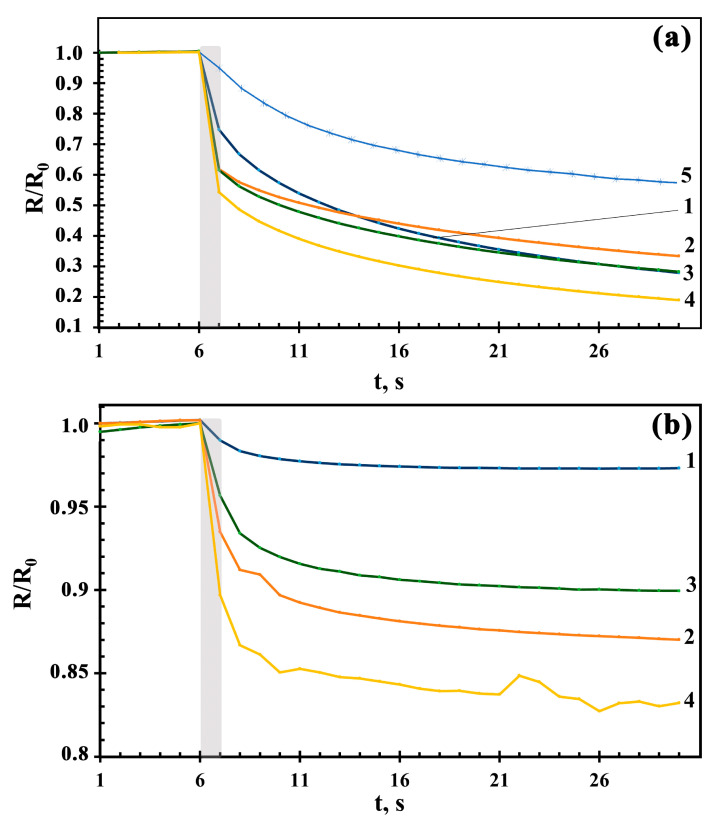
The photosensor time dependences of R/R_0_ for 1Co-99Zn (1), 3Co-97Zn (2), 5Co-95Zn (3), 10Co-90Zn (4), and ZnO (5) films when exposed to radiation with wavelengths of 400 nm (**a**) and 660 nm (**b**).

For all obtained films, the direct measurement of photoconductivity was carried out under the constant action of light from LEDs with peak wavelengths of 400 nm, 470 nm, 525 nm, 625 nm, 660 nm, and 940 nm [23]. It was noted that exposure to LED radiation with a wavelength of 940 nm did not lead to a response in any film. At the same time, the ZnO film reacted to radiation only at the wavelength of 400 nm. For other films, a decrease in resistance was observed due to a photogeneration of free charge carriers when exposed to radiation with a wavelength of 660 nm or less. The measurement of photoconductivity was carried out prior to the beginning of the dependences “flattening”. Figure 9 shows the dependences of the sensor resistance, R, normalized to the initial resistance, R_0_, when exposed to radiation with a wavelength of 400 nm and 660 nm for the studied films. Based on these dependences, the response time, t_res_, was determined, at which the film response was 90% of the maximum.

Studies have shown that the smallest values of the photoresponse are characteristic for the ZnO film (Figure 9a). With adding Co_3_O_4_, the amplitude of the photoresponse increases with higher cobalt oxide and reaches a maximum for the 10Co-90Zn film. In addition, the t_res_ value has rather large values, more than 10 s when exposed to an LED with the wavelength of 400 nm; these data are listed in Table 2. When exposed to an LED with a wavelength of 660 nm, t_res_ is slightly less: 6–9 s. Based on the dependences of R/R_0_(t), it can be noticed that exposure to radiation for films with a high content of Co_3_O_4_ has a clear, pronounced, sharp reduction in resistance during the first second. This is due to a fast photogeneration of nonequilibrium charge carriers. With longer exposure time, the observed changes in photosensitivity are slower. We suggest that this type of photoresponse appears from the presence of Co_3_O_4_ in the nanocomposite structure of Co_3_O_4_-ZnO films, which contributes to the fast generation of charge carriers.

According to the phenomenological theory of photoconductivity, charge carriers resulting from the photoionization are nonequilibrium ones. The generation of nonequilibrium charge carriers, electrons (Δ*n*), and holes (Δ*p*) leads primarily to a change in the conductivity of the semiconductor (σ), which for Co_3_O_4_-ZnO films should be written as Equation (3):

(3) $$\sigma =e\cdot \left(\mu_{n}\cdot n_{0}+\mu_{p}\cdot p_{0}+\mu_{n}\cdot \Delta n+\mu_{p}\cdot \Delta p\right)$$

where *e* is the charge of the electron, $n_{0}$ and $p_{0}$ are the equilibrium concentration of electrons and holes, respectively, and *µ_n_* and *µ_p_* are the mobility of electrons and holes, respectively.

Therefore, the excess (nonequilibrium) photoconductivity (Δ*σ*) can be written as Equation (4):

(4) $$\Delta \sigma =e\cdot \left(\mu_{n}\cdot \Delta n+\mu_{p}\cdot \Delta p\right)$$

In Co_3_O_4_-ZnO films, which have similar properties as intrinsic semiconductors, the slightest photogeneration of charge carriers will lead to a significant change in conductivity/resistivity. To check the response rate of the derived photosensors, measurements of the film photoconductivity were carried out under a pulsed exposure to LED radiation with wavelengths of 400 nm and 660 nm with a frequency of 5 Hz. To compare the parameters of sensors based on Co_3_O_4_-ZnO films, the normalized photoresponse (Δ*I_n_*) was calculated using Formula (5) [18,42]:

(5) $$\Delta I_{n}=\frac{I-I_{0}}{I_{0}}$$

**Figure 10:**
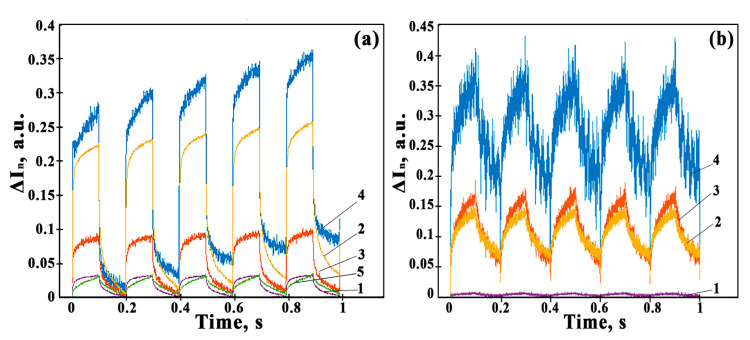
Normalized photoresponses of sensors based on 1Co-99Zn (1), 3Co-97Zn (2), 5Co-95Zn (3), 10Co-90Zn (4), and ZnO (5) films when exposed to radiation with wavelengths of 400 (**a**) and 660 nm (**b**) under pulses with a frequency of 5 Hz.

Figure 10 shows Δ*I_n_* values for sensors based on Co_3_O_4_-ZnO films with different concentrations of Co_3_O_4_. Figure 10 show that a reproducible photoresponse occurs when exposed to both wavelengths for all Co_3_O_4_-ZnO films, but its amplitude and time are varied for films with different concentrations of Co_3_O_4_. The maximum photoresponse when exposed to radiation with a wavelength of 400 nm is observed in a sensor based on a 10Co-90Zn film. The photoresponse of the sensor based on the 3Co-97Zn film turned out to be higher than that of the sensor based on the 5Co-95Zn film. As can be seen from Figure 6, this is due to the fact that the composite oxide is more depleted by charge carriers in the 3Co-97Zn film. Accordingly, the generation of nonequilibrium charge carriers due to the influence of radiation will lead to higher values of the photoresponse. For pure ZnO and 1Co-99Zn films, the photoresponse values were found to be almost the same. However, the response time of the 1Co-99Zn film is shorter.

From Figure 10, the response time, t*, was estimated for all films, where the response of the film was 90% of the maximum, and the average lifetime of nonequilibrium charge carriers, τ*, was calculated according to the Formula (6) [43]:

(6) $$\Delta I_{n}=1-e^{-\frac{t}{\tau^{*}}}$$

**Figure 11:**
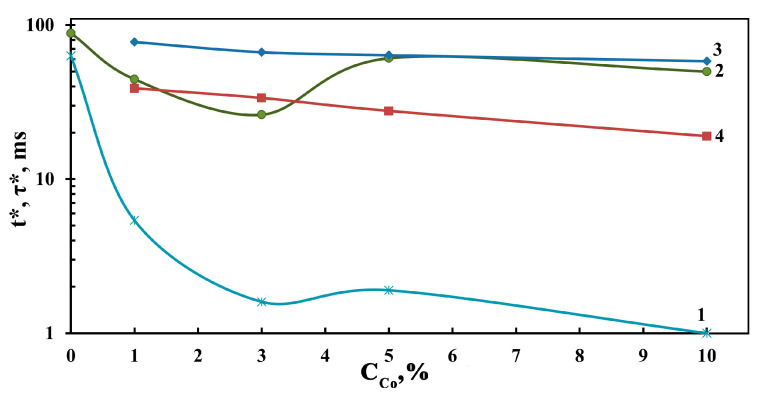
The dependences of the average lifetime of nonequilibrium charge carriers (1, 4) and the response time (2, 3) of photosensors based on Co_3_O_4_–ZnO films on the content of Co_3_O_4_ when exposed to LED radiations of 400 nm (1, 2) and 660 nm (3, 4).

The plotted dependences of t* and τ* on the concentration of Co_3_O_4_ in the composite films are collected in Figure 11. These data show that the t* value decreases from 88.5 ms, characteristic for the ZnO film, to 26.2 ms for the 3Co-97Zn film, and further increases to 50–60 ms for 10Co-90Zn and 5Co-95Zn. The values of τ* go down from 63 ms for the ZnO film to 5.4 ms for the 5Co-95Zn film and further to 1–2 ms for other films. When exposed to radiation with a wavelength of 660 nm, the values of t* decrease uniformly from 78 ms to 58 ms, while the values of τ* are reduced from 39 ms to 19 ms with an increase in the concentration of cobalt oxide in the Co_3_O_4_-ZnO films. This fact testifies to the undeniable effect of the Co_3_O_4_ additive on increasing the speed of radiation sensors based on Co_3_O_4_-ZnO films.

To compare our data with the literature, we listed available data in Table 3. As one can see, an increase in the content of Co_3_O_4_ in the composite films leads to higher-speed photosensors at the range of 400–660 nm wavelengths.

## 4. Conclusions

In this work, nanocomposite Co_3_O_4_–ZnO films with a cobalt content of 1–10 mol.% were obtained by solid-phase pyrolysis. The films consist of a ZnO wurtzite phase and a cubic structure of Co_3_O_4_ spinel. The crystallite size forming the film goes up from 18 nm to 24 nm with an increase in the Co_3_O_4_ content. The Co_3_O_4_ content up to 10 mol.% leads to a reduction in optical absorption at the visible light range up to 65%. At the same time, the band gap, evaluated from the Tauc plot to be 3.94–3.98 eV, shows the presence of local states with energies of 1.65 eV and higher. This is also confirmed by the estimation of the valence band maximum via the XPS method. In addition, an increase in the resistivity of films up to 10^4^ Ohm·cm and a decrease in the concentration of charge carriers to 10^12^ cm^−3^ indicate producing a material with a semiconductor conductivity close to intrinsic nature. The nanocomposite structure of the material where p-semiconductor (Co_3_O_4_) crystallites come into contact with n-semiconductor (ZnO) crystallites leads to evolving a high surface potential up to 487 mV, which in turn leads to the existence of a surface electric field with a strength reaching 10^5^–10^7^ V/cm values. The latter makes it possible to design high-speed photosensors based on 10Co-90Zn films for the range of 400–660 nm wavelengths with a response time of ca. 26–58 ms.

## Figures and Tables

**Table 1:** The content of element atoms in Co_3_O_4_-ZnO films based on XPS data.

Film Material	The Content of Element Atoms		
	O1s	Co2p	Zn2p
1Co-99Zn	50	1	49
3Co-97Zn	49	3	48
5Co-95Zn	49	5	46
10Co-90Zn	51	9	40

**Table 2:** Response time, t_res_, under a constant exposure to LED radiation with wavelengths of 400 nm and 660 nm.

Co_3_O_4_/ZnO-Films	t_res_ (s), 400 nm	t_res_ (s), 660 nm
ZnO	15.5	---
1Co-99Zn	14.5	6.5
3Co-97Zn	13.5	8.0
5Co-95Zn	13.0	8.5
10Co-90Zn	12.5	7.0

**Table 3:** Characteristics of response time for photosensors based on Co_3_O_4_-ZnO films or Co_3_O_4_-ZnO structures.

**Synthesis Method,** **Structure**	**The Optical Transmittance, %**	**Wavelength, nm**	**Response Time**	**Reference**
Reactive sputtering method, Co_3_O_4_-ZnO p-n- heterojunction	~76	White light	81.7 μs	[5]
Chemical growth method, Co_3_O_4_-ZnO composite structure	<8	Solar simulator	20 s	[44]
Spray pyrolysis, Co_3_O_4_ thin films	-	442 nm −633 nm	12 s 24 s	[18]
Sputtering method and hydrothermal synthesis, core–shell Co_3_O_4_-ZnO structures	<30	Visible light	6 s	[45]
Rapid liquid deposition, Co_3_O_4_-ZnO composite films (Co:Zn = 3:1)	-	335 nm	15 s	[19]
Solid-phase pyrolysis, 3Co-97Zn nanocomposite films, 10Co-90Zn nanocomposite films	>65	400 660	26.2 ms 58.3 ms	This work

## Data Availability

The data presented in this study are available on request from the corresponding author.

## Supplementary Materials

The following supporting information can be downloaded at: https://www.mdpi.com/article/10.3390/s23125617/s1, Figure S1: Distribution of ZnO (a,b) 1Co-99Zn (c,d), 3Co-97Zn (e,f), 5Co-95Zn (g,h), 10Co-90Zn (i,k) films surface potential.
