# Supplementary figures and images for: Nanocomposite Co3O4-ZnO Thin Films for Photoconductivity Sensors
Source: Sensors (Basel). 2023 Jun 15;23(12):5617. doi: 10.3390/s23125617 (PMC10305615; doi:10.3390/s23125617)

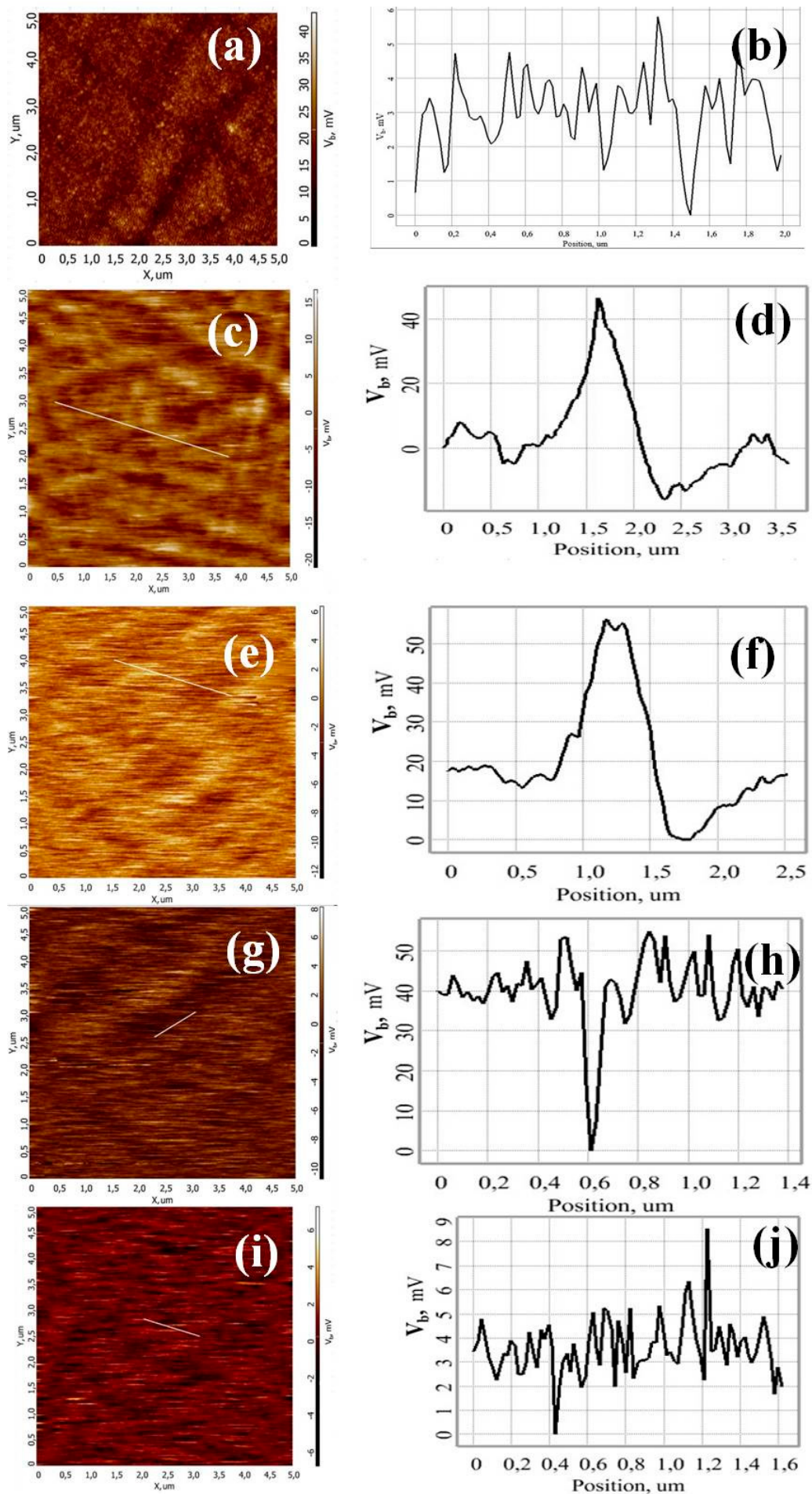

**Figure S1.** Distribution of ZnO (a,b) 1Co-99Zn (c,d), 3Co-97Zn (e,f), 5Co-95Zn (g,h), 10Co-90Zn (i,k) films surface potential.

Supplement: Supplementary file 1 [file sensors-23-05617-s001.zip › sensors-2440160-supplementary.pdf]
